# Parkinson’s disease speech production network as determined by graph-theoretical network analysis

**DOI:** 10.1162/netn_a_00310

**Published:** 2023-06-30

**Authors:** Jana Schill, Kristina Simonyan, Simon Lang, Christian Mathys, Christiane Thiel, Karsten Witt

**Affiliations:** Department of Neurology, School of Medicine and Health Sciences, University of Oldenburg, Oldenburg, Germany; Department of Otolaryngology, Head and Neck Surgery, Harvard Medical School, Boston, MA, USA; Department of Otolaryngology, Head and Neck Surgery, Massachusetts Eye and Ear, Boston, MA, USA; Institute of Radiology and Neuroradiology, Evangelisches Krankenhaus, University of Oldenburg, Oldenburg, Germany; Research Center Neurosensory Science, University of Oldenburg, Oldenburg, Germany; Department of Diagnostic and Interventional Radiology, University of Düsseldorf, Düsseldorf, Germany; Department of Psychology, School of Medicine and Health Sciences, University of Oldenburg, Oldenburg, Germany

**Keywords:** Parkinson’s disease, Speech production network, Functional magnetic resonance imaging, Network analysis, Emotional distraction

## Abstract

Parkinson’s disease (PD) can affect speech as well as emotion processing. We employ whole-brain graph-theoretical network analysis to determine how the speech-processing network (SPN) changes in PD, and assess its susceptibility to emotional distraction. Functional magnetic resonance images of 14 patients (aged 59.6 ± 10.1 years, 5 female) and 23 healthy controls (aged 64.1 ± 6.5 years, 12 female) were obtained during a picture-naming task. Pictures were supraliminally primed by face pictures showing either a neutral or an emotional expression. PD network metrics were significantly decreased (mean nodal degree, *p* < 0.0001; mean nodal strength, *p* < 0.0001; global network efficiency, *p* < 0.002; mean clustering coefficient, *p* < 0.0001), indicating an impairment of network integration and segregation. There was an absence of connector hubs in PD. Controls exhibited key network hubs located in the associative cortices, of which most were insusceptible to emotional distraction. The PD SPN had more key network hubs, which were more disorganized and shifted into auditory, sensory, and motor cortices after emotional distraction. The whole-brain SPN in PD undergoes changes that result in (a) decreased network integration and segregation, (b) a modularization of information flow within the network, and (c) the inclusion of primary and secondary cortical areas after emotional distraction.

## Author Summary

Parkinson’s disease (PD) features a variety of motor and nonmotor symptoms, which relate to changes in functional brain connectivity. This study investigated how the whole-brain speech production network (SPN) is affected by PD. The paradigm included an emotional distraction component to assess the SPN’s susceptibility to an emotional influence. The SPN was found to be less integrated and less segregated in PD than in healthy controls. Emotional distraction had a greater effect on the SPN in PD than in healthy controls. Key nodes for information flow (network pillars) were mainly located in associative cortices. They were robust against emotional distraction in healthy controls, but shifted to primary and secondary cortical areas in PD.

## INTRODUCTION

Parkinson’s disease (PD) is the second most common neurodegenerative disease (Tysnes & Storstein, 2017). Due to a predominant loss of dopaminergic cells in the substantia nigra (SN) and other brain regions, a cascade of alterations in brain structure and function takes place, leading to a variety of symptoms (Dickson, 2018; Obeso et al., 2000). While the causal relation between SN cell loss and the emergence of motor symptoms has been well researched (e.g., Foffani & Obeso, 2018; Frosini, Cosottini, Volterrani, & Ceravolo, 2017), the pathogenesis of PD nonmotor symptoms is more complex and elusive.

One class of symptoms of PD are speech difficulties, describing impairments of voice, fluency, and articulation (Ho, Iansek, Marigliani, Bradshaw, & Gates, 1998). The neurological basis of speech impairment in PD is not fully understood, as speech and language control is affected by both, motor symptoms as well as nonmotor symptoms. Speech production is a highly complex task involving perception, reasoning, emotional and cognitive skills, motor programming, as well as a proper motor output. In PD the precision control of muscle innervation necessary for a proper speech output is affected, reflecting impaired motor performance. Language and speech control is furthermore affected by nonmotor symptoms related to disturbances in emotion regulation, mood changes and cognitive difficulties as well as drawling and salivation (Critchley, 1981; Jankovic, 2008). The targeted examination of whole-brain functional brain networks and their changes has proven useful in studying and understanding natural language formation (Fuertinger, Horwitz, & Simonyan, 2015) as well as its changes in other movement disorders (Fuertinger & Simonyan, 2017). As PD has been linked to changes within network structures due to different pathophysiological mechanisms, it is likely the speech production network (SPN) is altered in PD. However, it has not been investigated in detail whether this is indeed the case.

In recent years, more and more disorders have been attributed to changes in brain network function (Ko, Spetsieris, & Eidelberg, 2017; Palop & Mucke, 2016; Pini et al., 2020; Schirinzi, Sciamanna, Mercuri, & Pisani, 2018). Instead of investigating the role of a specific set of brain regions for a task as measured by their individual activation patterns, network analysis looks at the interplay of these regions (Bullmore & Sporns, 2009). Given the multifactorial origin of PD symptoms driven by changes in multiple neurotransmitter systems and a heterogeneous spread of pathology in the course of the disease, network analysis seems to be specifically fruitful for revealing changes in PD brain network control. Graph-theoretical network analysis is one category of network analyses that considers the different regions of the brain as nodes of a graph and defines the edges of the graph by measures of association between these brain regions (Bullmore & Sporns, 2009). Thereby, mathematical graph computations can be used to characterize the functional connectivity of the brain. Network integration (the network’s ability to propagate information efficiently throughout the network) and network segregation (the network’s ability for specialized processing within densely interconnected groups of nodes, i.e., clusters) provide insight into the workings of a network and therefore, in the case of brain network analyses, shed light on how the brain processes information (Rubinov & Sporns, 2010). Another approach for investigating network function is a hub analysis (van den Heuvel & Sporns, 2013). Here, nodes that contribute most to the network are identified as hubs and then analyzed in terms of their spatial distribution throughout the network and their communication patterns with other nodes. By assigning highly interconnected groups of nodes into different network modules, the modularity of the network can be assessed. Hubs that connect different modules (connector hubs) facilitate network integration, whereas hubs that are dominantly connected to nodes of their own module facilitate network segregation.

Network analyses have revealed changes in integration and segregation associated with PD. In an MRI resting-state study with drug-naïve PD patients, Luo et al. (2015) found that while network integration was unchanged, patients showed reduced functional segregation as compared to healthy controls, which they interpret as the PD brain network being more random and less optimally organized. Reduced functional segregation was also reported by Kim et al. (2017) in a study on MRI brain network dynamics in PD patients. A longitudinal MEG study in PD patients revealed that while network integration was not different from controls in de novo patients, it decreased with disease progression (Olde Dubbelink et al., 2014). Functional segregation, however, was reduced right from the beginning. Taken together, these studies indicate that resting-state network integration and segregation are affected differently by PD. However, to our knowledge there are no studies investigating whole-brain functional connectivity during speech and emotion processing. Furthermore, the analysis of resting-state network hubs revealed that PD affects hub function and organization (Koshimori et al., 2016). Here, we extend this research to task-based networks.

In clinical practice, cognitive and emotional challenges are often accompanied by a worsening of motor symptoms in PD, best seen in an increase of tremor amplitude in a cognitive or an emotional task (Raethjen et al., 2008). This emotional–motor interaction demonstrates the influence of a secondary task on motor performance, which has been documented also in dual-task situations during gait (Raffegeau et al., 2019). In the present study we challenge the SPN by a distractor task to assess how speech production is affected by an emotional challenge. Emotion processing is a nonmotor domain that is affected by PD, and an impairment in recognizing and discriminating emotions based on facial cues has been described (Aiello et al., 2014; Castner et al., 2007; Iyer, Au, Angwin, Copland, & Dissanayaka, 2019; Wagenbreth et al., 2019). Therefore, a detailed assessment of the emotional prime is important to evaluate the conscious recognition of the emotional distractor.

In this study, we investigated the whole-brain functional networks of PD patients and elderly healthy controls solving a picture-naming task primed with affective face stimuli. The facial primes were intended to redirect processing resources toward the emotional distraction, without necessarily inducing an emotion in participants. We analyzed the brain network topology in terms of its segregation and integration and investigated communication patterns within the network by a hub analysis. Furthermore, we illustrated which regions carry the communicative load of the network by looking at network pillars, that is, exceptionally well connected hubs (Schill et al., 2023). We assessed how robust they were against emotional challenge and neurodegeneration as present in PD. We answered two main research questions: First, we determined how the SPN is affected by PD. Second, we assessed how emotional distraction influenced the SPN and how this influence was affected by PD.

Our hypotheses were mainly based on resting-state network studies. We assumed that resting-state findings would translate into the SPN: We expected network integration in the SPN of PD patients to be similar to that of controls, while network segregation should be decreased. Furthermore, a hub analysis was performed to assess differences in the distribution of key nodes of information processing between modules (connector hubs) and within a given module (provincial hubs). Lastly, we explored how the network changed after presentation of an emotional distractor. We expected the PD patients’ network to be more susceptible to emotional distraction, given the emotional–motor interference clinically evident in the everyday life of patients.

## METHODS

### Participants

Nineteen patients with PD and 25 age-matched healthy volunteers were recruited in this study. The study only included right-handed native German speakers. Participants did not suffer from neurological and/or psychiatric diseases (other than PD in patients) or speech disorders and did not take psychoactive drugs (medical or recreational). Diagnosis of PD was established by a neurologist according to the medical history and the MDS-Clinical Diagnose Criteria for PD (Postuma et al., 2015). Patients were on their usual medication scheme during the study (levodopa equivalent dose: 539.2 mg ± 319.9 mg). The short form of the Edinburgh Handedness Inventory (Veale, 2014) was used to verify handedness (a score of >60 classified the participant as right-handed). To exclude dementia the Montreal Cognitive Assessment (MoCA; Nasreddine et al., 2005) with a cutoff score of >23 was applied (scores range from 0 to 30, with higher scores indicating better cognitive performance). Beck Depression Inventory II (Beck, Steer, & Brown, 1996) with a cutoff score of <20 was used to establish the absence of depressive symptoms (scores range from 0 to 63, with higher scores indicating more depressive burden).

Seven participants had to be excluded from the analysis for the following reasons: motion artifacts (1), other imaging artifacts (1), misunderstood task (1), structural brain abnormality (1), depressive disorder (1), and abnormal mean connectivity strength (2), determined by an outlier analysis using Tukey’s fences (Tukey, 1977). Therefore, 23 healthy volunteers (64.1 ± 6.5 years of age, 12 females/11 males) and 14 patients (59.6 ± 10.1 years of age, 5 females/9 males) were included in the final analysis. Groups were matched in age and years of education (see Table 1 for demographic characteristics). Data from healthy volunteers had previously been analyzed in a study on healthy aging (Schill et al., 2023).

**Table 1.** Group demographics and clinical characteristics of PD patients and controls

Characteristic	PD patients *n* = 14	Controls *n* = 23	*P*
Age (years)	59.6 ± 10.1	64.1 ± 6.5	0.15
Sex (female/male)	5/9	12/11	0.33
Education (years)	17.96 ± 6.22	17.65 ± 4.0	0.85
Montreal Cognitive Assessment	27.71 ± 1.94	28.39 ± 1.27	0.21
Average head motion (relative)	0.15 ± 0.06	0.14 ± 0.04	0.79
PD duration (months)	34.29 ± 27.90	–	–
MDS-UPDRS part III, motor exam	15.64 ± 9.39	–	–
Hoehn and Yahr stage	1.29 ± 0.73	–	–

*Note*. Statistical significance was assessed by unpaired *t* tests, except for gender, for which a *χ*^2^ test was used.

All participants gave their written informed consent. This study was approved by the local ethics committee of the University of Oldenburg and was conducted in accordance with the Declaration of Helsinki.

### Experimental Paradigm

**Figure 1.**
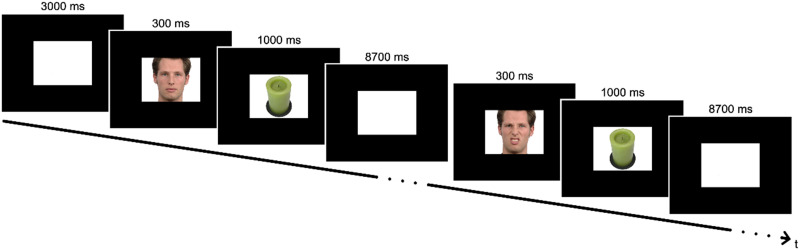
Experimental paradigm. Per run, 30 objects were shown. Each object appeared twice, once primed with the neutral und once primed with the disgusted version of the same face. Faces were shown for 300 ms, objects for 1,000 ms. During the interstimulus interval of 8,700 ms, a white background was shown.

Participants solved a picture-naming task in the MRI scanner, which has been described previously (Schill et al., 2023). Participants were shown a total of 60 everyday inanimate objects, which they had to name correctly (30 tasks per run). Each picture was preceded by a presentation of a priming facial picture. The primes were either emotionally neutral (neutral condition) or showed a disgusted expression (disgusted condition). Object pictures were taken from the BOSS database (Brodeur, Guérard, & Bouras, 2014), while primes were obtained from the Radboud Faces Database (Langner et al., 2010). Faces were balanced between gender (15 male, 15 female) and pseudorandomly matched with objects to ensure that each object was paired with the neutral and disgusted version of the same face. The experiment was split in two runs. In each run, 30 objects appeared twice (once primed with a neutral face and one with a disgusted face). Primes appeared for 300 ms, immediately followed by 1,000 ms of object presentation. Since conscious recognition of emotional face expressions was desired, we conducted a pilot study in which a presentation duration of 300 ms for recognition of emotional facial expression was shown to be sufficient for patients. The interstimulus interval was set to 8,700 ms, in which a white background was shown (Figure 1). For audio analysis, participants solved the same task in an acoustic chamber after the MRI scan. To ensure that performance was comparable between scanner and acoustic chamber, the number of correct answers was determined in both settings. Controls’ performance was 98.9% in the MRI and 98.3% in the acoustic chamber. Patients scored 96.4% in the MRI and 96.5% in the acoustic chamber. For further MRI analysis, only correct answers were considered. After MRI and acoustic chamber sessions, participants had to rate all facial primes on a scale from 1 (negative emotional expression) to 9 (positive emotional expression). These emotional scores were used to assess group differences in perceived emotional valence of the facial stimuli.

### Behavioral Analysis

For all speech recordings obtained in the acoustic chamber, speech onset was automatically determined using Chronset (Roux, Armstrong, & Carreiras, 2017). Audio intensity was obtained using Praat (Boersma, 2001). The mean and maximum intensity was calculated for all recordings, excluding the recording time before speech onset. Speech onset, mean, and maximum intensity were averaged for each person for each condition separately. For each condition, emotional scores of the facial primes were averaged. These average scores were compared between groups. A mixed analysis of variance (ANOVA), with group as between-subject factor and emotion (neutral, disgusted) as within-subject factor, was employed for both metrics (speech onset and emotional scores) separately (*p* < 0.5).

### Image Acquisition

Structural and functional scans were obtained at a 3T whole-body MRI scanner (Siemens Magnetom Prisma, Siemens Healthyneers, Erlangen, Germany) with a 64-channel head coil. Stimulus presentation was done in Cogent 2000 (https://www.vislab.ucl.ac.uk/cogent.php, RRID: SCR_015672). Three-dimensional T1-weighted scans using a magnetization-prepared rapid gradient-echo (MPRAGE) sequence were used to obtain structural images. Parameters were TR = 2 s, TE = 2.07 ms, TI = 952 ms, 1 volume, 224 slices, slice thickness = 0.75 mm, FOV = 240 mm × 240 mm, flip angle = 9°, voxel size = 0.75 × 0.75 × 0.75 mm^3^. Functional scans were obtained via a whole-brain echo planar imaging (EPI) sequence with the following parameters: TR = 2 s, TE = 30 ms, 310 volumes, 36 slices, slice thickness = 3 mm, FOV = 192 mm × 192 mm, flip angle = 75°, voxel size = 3 × 3 × 3 mm^3^.

### Task Activation Analysis

For quality control regarding our paradigm, we employed a general linear modeling approach to assess which kind of activation was elicited by the emotionally primed picture-naming task. Further information can be found in the Supporting Information.

### Functional Network Construction

The network construction and analysis pipeline has been described previously (Schill et al., 2023). Structural images were skull stripped with ANTs software (Avants et al., 2011). The remaining preprocessing steps were done in AFNI (Cox, 1996) using afni_proc.py: The first four volumes of the functional scans were removed to assure signal stability. Then, the scans were despiked using default settings and a Fourier interpolation was used to temporally align them to the beginning of the TR. They were spatially aligned to the first volume. Then they were normalized to the AFNI standard Talairach-Tournoux space (nonlinear). Spatial smoothing was done using a 4-mm full width at half maximum Gaussian kernel. In a last step, the voxel time series were scaled such that their mean was set to 100. Thereby, they were normalized to their percent signal change, ensuring comparability between scans. TRs with motion artifacts above 0.5 mm were excluded from further analysis. They were removed from the dataset without interpolation. All participants had less than 15% of TRs affected. Average relative head motion did not differ between groups (Table 1). The resultant preprocessed images were the basis for the network analysis.

A total of 212 regions of interest (ROIs) were included in the analysis. In order to fully cover the entire brain, 142 cortical, 36 subcortical, and 34 cerebellar ROIs were defined by masks obtained from the cytoarchitectonic probability maps and macrolabel atlas (Eickhoff et al., 2005; Fuertinger et al., 2015). The fMRI signal was used to create condition-specific voxel time series for every participant. All trials containing a neutral prime were concatenated to yield the neutral-condition voxel time series. This was done accordingly for the disgusted condition. To avoid signal spillover due to the randomized event-related design, we decided to include only four TRs of every trial, starting with the first TR after the object appeared on the screen. The condition-specific voxel time series were then averaged within each ROI for every participant separately. Then, individual connectivity matrices were constructed by calculating normalized mutual information (NMI) coefficients of ROI pairs. We chose NMI coefficients instead of Pearson’s correlation because they have the inherent advantage of being non-negative, but preserve the nonzero structure of the Pearson’s correlation (see Fuertinger & Simonyan, 2017, for a discussion on NMI vs. Pearson’s correlation). NMI coefficients were calculated by dividing the classical mutual information (Cover & Thomas, 2012) by the geometric mean of the associated Shannon entropies (Shannon, 1948). Therefore, the NMI measures statistical dependence on a scale from 0 (independence) to 1 (mutual dependence).

Participants’ condition-specific connectivity matrices were group averaged, yielding two matrices per group (one neutral and one disgusted). Based on these group matrices, weighted undirected graphs were created, in which the ROIs served as nodes and the NMI coefficients served as edge weights. Participants with an abnormal mean connectivity strength as determined by Tukey’s fences (Tukey, 1977) were excluded from the respective condition-specific group matrix.

Network density was calculated by dividing the number of nonzero edges by the number of possible connections. To avoid random graph characteristics due to high density (Humphries, Gurney, & Prescott, 2006; Lynall et al., 2010), percolation thresholding was applied as described by Bordier, Nicolini, and Bifone (2017). We used this thresholding method as it has been shown to be an appropriate choice for case-control studies (Bordier et al., 2017). Network construction was done using Python 3.7 and its open source libraries NumPy (Oliphant, 2015), SciPy (Virtanen et al., 2020), and Matplotlib (Hunter, 2007).

### Graph-Theoretical Network Analysis

For each condition separately, the two groups’ networks were compared. Furthermore, condition differences within each group were assessed. Five *network metrics* were computed: network density, mean nodal degree, mean nodal strength, mean clustering coefficient, and global efficiency. These were compared between networks using permutation *t* tests with 20,000 randomizations (*p* < 0.05; Bonferroni-corrected for multiple comparisons: *p* < 0.002). Permutation tests are nonparametric, as they are independent of the underlying distribution. This makes them very suitable for network analysis, as the distribution of test statistics is often not known in this field. The *mean nodal degree* is the average of all nodal degrees, which is the node’s number of nonzero connections. Analogously, the *mean nodal strength* is the average of all nodal strengths. Nodal strength is the sum of weights of each node’s edges. The two measures were normalized by dividing them through the number of network nodes. They both measure how well the network is connected and can be considered measures of network integration. The *mean clustering coefficient* is the average of all nodal clustering coefficients. The clustering coefficient of a node is the geometric mean of weights in triangles around the node. It shows the presence of functional cliques around the node, and can thereby be considered a measure of nodal segregation. *Global efficiency* describes how well information can be propagated throughout the network and is therefore a measure of network integration. It was calculated as the average inverse shortest path length. Path length was calculated as the sum of the inverse edge weights of all edges belonging to a path.

The *optimal modular decomposition* of each network was determined by maximizing the Newman modularity (Newman, 2006). For this, a heuristic optimization strategy using the Kernighan–Lin algorithm (Sun, Danila, Josić, & Bassler, 2009) was employed. This means that modules were determined by maximizing the number of intramodule edges and minimizing the number of intermodule edges. Each node was first assigned its own module. Then, the optimization algorithm was run 100 times to account for randomness (Fuertinger et al., 2015). As the initial modular assignment was random, we used a permutation approach to ensure maximal modular overlap between groups and conditions. As a measure of normalized variation of information between two modular assignment vectors, the partition distance (Meilă, 2007) was used to establish differences in optimal modular decompositions between groups and conditions. Further analysis steps based on modular decomposition always employed the decomposition of the specific network in question.

Moreover, we assessed *hub formation*: Nodes with a nodal degree or nodal strength more than 1 standard deviation (*SD*) above the mean were considered hubs. Nodes within the top 30% of nodal degree and nodal strength that did not meet the hub criterion were considered high-influence nodes, as they were still highly connected. Hubs were classified as connector hubs if they facilitated communication between different modules, or as provincial hubs if they facilitated communication within their module. This differentiation was made based on the nodal participation coefficient (van den Heuvel & Sporns, 2011), which is a measure of the distribution of inter- versus intramodule connections. As a cutoff value we chose 10% of the maximum nodal participation coefficient, with hubs passing the cutoff being classified as connector hubs and all others as provincial hubs. Hub type and spatial distribution were used as measures of comparison between groups and conditions. Differences in hub formation were assessed by a χ^2^ test of homogeneity (*p* < 0.05) concerning the frequencies of the different categories (connector hub, provincial hub, high-influence node, normal node). In a next step, we determined *network pillars* (Schill et al., 2023). With this term, we refer to hubs that play a key role in propagating information throughout the network. We consider hubs as network pillars if at least one of the following is true: (a) their nodal degree was at least 1.5 *SD* above the mean nodal degree of their network, or (b) their nodal strength was at least 1.5 *SD* above the mean nodal strength of their network. The cutoff of 1.5 *SD* was chosen to ensure that the resulting pillars make an extraordinary contribution to network functioning due to their exceptional number of edges or their unusual strength of connections with other nodes. By visual inspection of the dataset we ruled out a higher cutoff of 2 *SD*, as this would have eliminated any pillars in the control networks. According to their key position within a network, we assume that network pillars should be located in cortical associative areas of information integration. Given their exclusive connections within a network, we hypothesize that pillars should stay in a stable localization regardless of disease-related changes or emotional distractors to ensure information integration in a complex task. We assessed whether the number and location of network pillars demonstrated disease-related changes or changes in the course of emotional load within the SPN.

Analysis scripts were mainly written using Python 3.7 and its aforementioned libraries. MATLAB (The MathWorks, Inc.) and the Brain Connectivity Toolbox (Rubinov & Sporns, 2010) were used to compute the optimal modular decomposition and the network metrics. The BrainNet Viewer (Xia, Wang, & He, 2013) was used to illustrate brain networks.

### Data and Code Availability

The data used in this study will be made available upon request under the conditions of a formal data sharing agreement. Analysis scripts have been used in a previous project and can be found online at https://github.com/simonyanlab/NetworkAnalysis_Speech_HealthyAging (Schill, Fuertinger, & Simonyan, 2022).

## RESULTS

### Behavioral Results

Speech onset did not differ significantly between groups (*F*_1,35_ = 1.32, *p* = 0.26) nor conditions (*F*_1,35_ = 0.41, *p* = 0.53). There was also no interaction effect (*F*_1,35_ = 0.36, *p* = 0.55). The same was true for mean speech intensity (group effect: *F*_1,35_ = 0.43, *p* = 0.52; condition effect: *F*_1,35_ = 2.15, *p* = 0.15; interaction effect: *F*_1,35_ = 0.001, *p* = 0.98), and maximum speech intensity (group effect: *F*_1,35_ = 3.94, *p* = 0.06; condition effect: *F*_1,35_ = 0.12, *p* = 0.73; interaction effect: *F*_1,35_ = 0.17, *p* = 0.68). Disgusted facial primes (*M* = 2.62, *SD* = 0.84) were rated significantly more negative than neutral primes (*M* = 5.08, *SD* = 0.55, *F*_1,35_ = 346.62, *p* < 0.0001). There was no significant group difference in emotional scores (*F*_1,35_ = 0.16, *p* = 0.69) and no interaction effect (*F*_1,35_ = 0.02, *p* = 0.89). Hence, basic parameters of speech production and evaluation of the emotional value are comparable between PD patients and healthy elderly participants.

### Network Metrics

Overall, PD patients demonstrated significantly decreased global network efficiency compared to controls. Measures of network integration (global efficiency, mean nodal degree, and mean nodal strength) and network segregation (mean clustering coefficient) showed significantly lower scores in PD patients. All metric values and respective statistical results are shown in Table 2. In contrast to healthy participants, in PD patients emotional distraction lead to significantly increased mean nodal degree and mean nodal strength, but these network metrics were still significantly lower than in healthy participants. In conclusion, compared to healthy controls, PD patients’ SPN demonstrated lower scores for information integration and segregation simultaneously. However, the PD patients’ metrics were more affected by emotional distraction than were the healthy controls’.

**Table 2.** Network metrics

		Controls	Patients	*P*
Network density	Neutral	49.08%	24.55%	0.209
	Disgusted	49.61%	32.56%	0.351
	*P*	0.967	0.218	
Mean nodal degree	Neutral	0.489 ± 0.229	0.244 ± 0.127	**
	Disgusted	0.494 ± 0.229	0.324 ± 0.165	**
	*P*	0.818	**	
Mean nodal strength	Neutral	0.072 ± 0.036	0.035± 0.018	**
	Disgusted	0.075 ± 0.037	0.044 ± 0.023	**
	*P*	0.487	**	
Mean clustering coefficient	Neutral	0.117 ± 0.021	0.096 ± 0.024	**
	Disgusted	0.121 ± 0.019	0.096 ± 0.020	**
	*P*	0.044	0.760	
Global efficiency	Neutral	0.110	0.086	*
	Disgusted	0.113	0.090	**
	*P*	0.616	0.050	

*Note*. Mean values are given ±1 standard deviation. *P* values were determined by a permutation *t* test with 20,000 repetitions.

* *p* < 0.002.

** *p* < 0.0001.

### Network Communication Patterns

**Figure 2.**
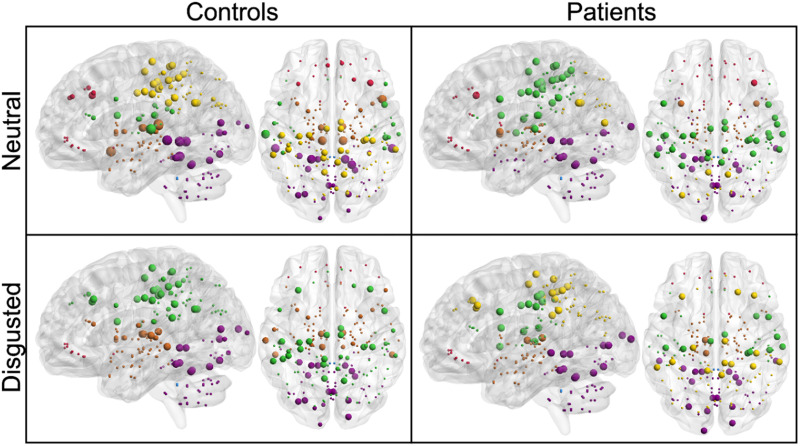
Optimal modular decomposition and hub localization. Module affiliation is denoted by color: purple = occipital-cerebellar module; brown = subcortical module; blue = cerebellar module; red = frontal module; green and yellow = parietal modules. Size denotes from largest to smallest: connector hubs, provincial hubs, high-influence nodes, other nodes. Module affiliation does not differ significantly between groups nor condition. Hubs differ in their characteristics and location.

There were no significant group or condition differences concerning the optimal modular decomposition of the whole-brain SPN as measured by the partition distance (see Table 3). The control group had six modules in the neutral condition and five in the disgusted condition. In both conditions there was an occipital-cerebellar module, a subcortical module, a very small cerebellar module, and a frontal module. In the disgusted condition, the fifth module was a parietal one. In the neutral condition, the parietal module was split in two separate modules. In the patient group, there were six modules in both conditions. Module affiliation was very similar to the controls’ SPN in the neutral condition, with the only differences appearing in the parietal regions. Module affiliation is depicted in Figure 2 for both groups and conditions.

**Table 3.** Partition distances of module affiliations

Comparison		Partition distance	*P*
Controls neutral	Patients neutral	0.172	0.254
Controls disgusted	Patients disgusted	0.134	0.624
Controls neutral	Controls disgusted	0.132	0.497
Patients neutral	Patients disgusted	0.196	0.217

**Figure 3.**
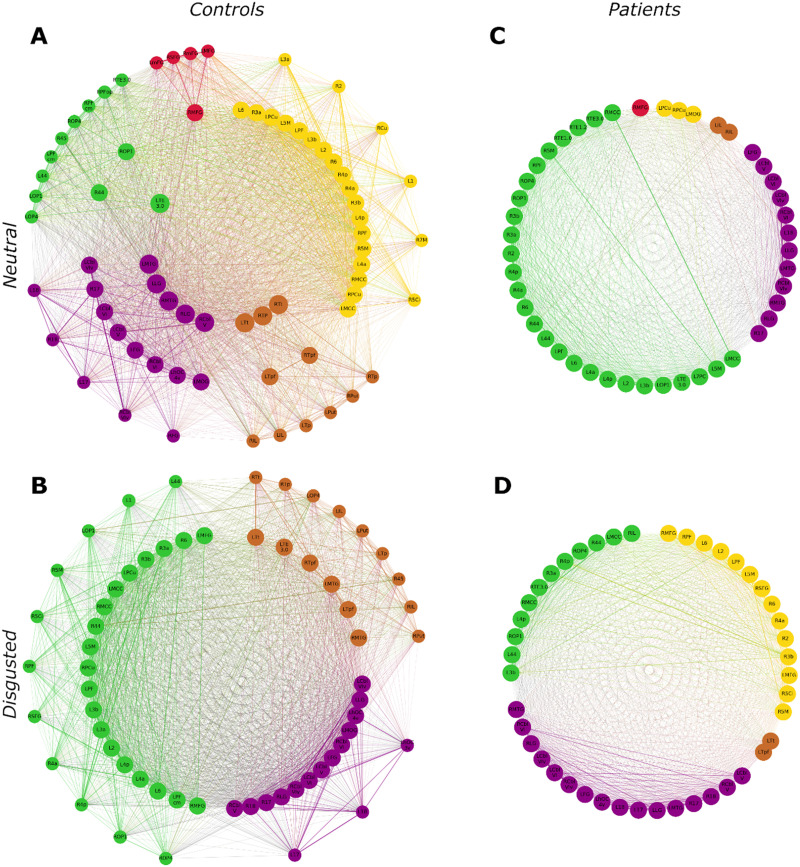
Hub characteristics. Hub formation is shown for (A) control group in the neutral condition, (B) control group in the disgusted condition, (C) patient group in the neutral condition, and (D) patient group in the disgusted condition. Nodes are arranged in three circles: Connector hubs are placed on the innermost circle, provincial hubs on the middle circle and high-influence nodes on the outer circle. Colors denote module affiliation. R = right; L = left; 1/2/3a/3b/6/17/18/44/45 = Brodmann areas (BA) 1/2/3a/3b/6/17/18/44/45; 4a/4p = anterior/posterior part of BA 4; 5Ci/5M = subdivisions of BA 5; 7M/7PC = subdivision of BA 7; Cbl-V/VI/VIv = cerebellar lobules V/VI/VI vermis; Cu = cuneus; FG = fusiform gyrus; hOC3v/hOC4v = ventral part of area hOC3/hOC4; IL = insula; LG = lingual gyrus; MCC = middle cingulate cortex; mFG = medial frontal gyrus; MFG = middle frontal gyrus; MOG = middle occipital gyrus; MTG = middle temporal gyrus; OP1/OP4 = operculum; PCu = precuneus; PF/PFcm/PFop = area PF/PFcm/PFop in the inferior parietal cortex; Put = Putamen; SFG = superior frontal gyrus; TE1.0/1.2/3.0 = area TE1.0/1.2/3.0 in the auditory cortex; Tp/Tpf/Tt = parietal/prefrontal/temporal subdivisions of the thalamus; TP = temporal pole.

Communication patterns differed between groups and conditions (Figure 3). Controls showed connector hubs in three different modules in the neutral condition, but no connector hubs in the disgusted condition. Patients, however, did not show any connector hubs, irrespective of the condition. Concerning the provincial hubs, controls showed an increase in the disgusted condition (31 vs. 38). Patients had nearly the same number of provincial hubs in both conditions (45 vs. 44) and had more provincial hubs than healthy controls did. Controls had more high-influence nodes in the neutral condition (30 vs. 23), while patients did not exhibit any high-influence nodes. Hub formation differed significantly between controls and patients in the neutral condition (*χ*^2^ = 43.6, *df* = 3, *p* < 0.0001). While hub formation differed significantly between conditions in the control group (*χ*^2^ = 10.9, *df* = 3, *p* < 0.01), such a condition effect was not present in the patient group (*χ*^2^ = 0.01, *df* = 1, *p* = 1). Taken together, patients’ intermodular communication was strongly diminished, while intramodular communication was increased.

**Figure 4.**
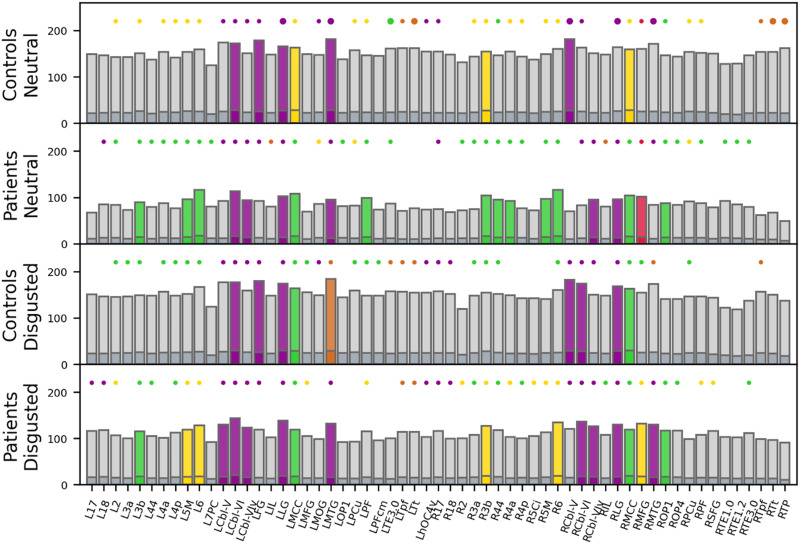
Network pillars. Pillars (colored bars) are shown for each group in each condition separately. Bars represent nodes that reached hub status in at least one of the four networks. Light grays bars = nodal degree. Dark gray bars = nodal strength. Circles indicate whether a node was a connector hub (large circle), a provincial hub (small circle), or no hub (no circle) in the respective network. Color of circles indicates which module a hub belongs to. Color of bars indicates pillar status: light/dark grey = no pillar; colored (according to module color) = pillar. R = right; L = left; 2/3a/3b/6/17/18/44 – Brodmann areas (BA) 2/3a/3b/6/17/18/44; 4a/4p = anterior/posterior part of BA 4; 5Ci/5M = subdivisions of BA 5; 7PC = subdivision of BA 7; Cbl-V/VI/VIv = cerebellar lobules V/VI/VI vermis; FG = fusiform gyrus; hOC4v = ventral part of area hOC4; IL = insula; LG = lingual gyrus; MCC = middle cingulate cortex; MFG = middle frontal gyrus; MOG = middle occipital gyrus; MTG = middle temporal gyrus; OP1/OP4 = operculum; PCu = precuneus; PF/PFcm = area PF/PFcm in the inferior parietal cortex; SFG = superior frontal gyrus; TE1.0/1.2/3.0 = area TE1.0/1.2/3.0 in the auditory cortex; Tpf/Tt = prefrontal/temporal subdivisions of the thalamus; TP = temporal pole.

In the healthy controls’ SPN, eight regions reached pillar status in the neutral condition: left cerebellar lobule VI, left fusiform gyrus, left lingual gyrus, left and right middle cingulate cortex, left middle temporal gyrus, right area 3b, and right cerebellar lobule V (Figure 4). The majority of these pillars was located in associative areas known to be involved in information integration from different sensory modalities, attention, and memory. Patients had more than twice as many network pillars. In addition to six regions that were pillars in the control network, they had pillars in left area 3b, bilateral area 5M, bilateral area 6, bilateral cerebellar lobule VIv, left auditory area prefrontal (PF), right area 44, right area 4a, right lingual gyrus, right middle frontal gyrus, and right operculum. This means that in the patients’ SPN, the communicative load was more distributed across the brain than in healthy controls.

In the controls’ SPN, pillars were robust against the emotional challenge. In the disgusted condition, seven of the eight neutral pillars remained. Only right area 3b lost pillar status, while two new regions became pillars, namely right cerebellar lobule VIv and right lingual gyrus. In the patients’ SPN, four regions lost pillar status after the emotional challenge (left auditory area PF, right area 44, right area 4a, right area 5M), while three new regions became pillars (left cerebellar lobule V, right cerebellar lobule VI, right middle temporal gyrus). In conclusion, patients exhibited more network pillars than controls. While pillars in the controls’ SPN were located in task-related areas, pillars in the patients’ SPN could also be found in auditory, sensory, and motor regions less obviously connected to the task.

## DISCUSSION

We found that the PD SPN differs from that of healthy controls in three main aspects: First, intermodular network communication was impaired in PD, as seen by a total absence of connector hubs. Second, while network pillars, that is, nodes with the most crucial role for information flow within the network, were predominantly located in associative cortices in healthy individuals, they were more widely distributed across the brain in PD patients. Third, the PD network was more susceptible to an emotional challenge than the control network, as seen by changes in network metrics and redistribution of network pillars. These differences in the SPN between PD patients and healthy participants are not accompanied by behavioral changes as measured here.

PD patients differed significantly from healthy controls in terms of network metrics. Both measurements of network integration (mean nodal degree, mean nodal strength, global efficiency) and segregation (mean clustering coefficient) scored below the metrics of healthy controls. The patient network was therefore less efficient and less organized. This study confirms the finding of previous studies analyzing resting-state networks in PD (Göttlich et al., 2013; Kim et al., 2017; Olde Dubbelink et al., 2014; Skidmore et al., 2011). The present study extended this observation to an active speech-processing task and furthermore demonstrated a significant change in certain PD network metrics after an emotional challenge toward a more organized network (but still significantly altered compared to healthy controls).

Our network communication analysis showed that in the neutral condition, the PD network displayed more provincial hubs than the control network, in the absence of both connector hubs and high-influence nodes. This suggests a breakdown of intermodular communication, indicating increased network segregation. Interestingly, network segregation as measured by the mean clustering coefficient was reduced in the patient network. One possible explanation could be that in the healthy networks, information processing was achieved via bundled pathways, with connector hubs acting as relay stations between modules. Within modules, nodes communicated mostly with their neighbors, leading to functional clustering. In the patient network, connector hubs were absent and could not act as intermodular relay stations. Therefore, intermodular communication must have been more distributed among all network nodes. This would in turn decrease network segregation, as nodes did not communicate in distinct clusters, but rather in a widespread manner. This interpretation favors a more random brain network organization, which has been reported for PD in the MEG domain (Olde Dubbelink et al., 2014).

In the healthy network, eight regions acted as network pillars, meaning that they played a key communicative role within the network. They were mostly located in association cortices, underlining their importance for higher order information processing and can directly be linked to speech or emotion aspects of the paradigm: The middle cingulate cortex, which bilaterally reached pillar status, is known to be involved in social cognition and integration of emotion processing and motor signals (Apps, Lockwood, & Balsters, 2013). Importantly, it has functional connections to premotor, frontal, parietal, and subcortical regions (Hoffstaedter et al., 2014), which presumably facilitated its status as pillar. The cerebellum, which also was found bilaterally to act as a network pillar, has been reported to be involved in emotion perception (Adamaszek et al., 2017) and word retrieval (De Smet, Paquier, Verhoeven, & Mariën, 2013). Furthermore, the left fusiform gyrus, lingual gyrus, and medial temporal gyrus reached pillar status. The fusiform gyrus plays a role on object recognition and face perception (Weiner & Zilles, 2016), while the lingual gyrus is implicated in processing facial expressions (Kesler-West et al., 2001; Kitada, Johnsrude, Kochiyama, & Lederman, 2010). Activation of the medial temporal gyrus has been reported during facial and language processing (Xu et al., 2015). Taken together, in the control network, the communicative load was distributed among several task-relevant regions. In the patient network, there were 11 more pillars. In addition to six regions that were pillars in the control network, the patient network had pillars in further auditory, sensory, and motor regions that were less obviously linked to the task. This is in line with our assumption that the patient network was less well organized and had a more random processing pattern and may need additional pillars to compensate information processing.

Our secondary aim was to assess how emotional distraction influences the variability of the SPN. Our behavioral analysis showed that disgusted faces were perceived as more negative than neutral faces, although this difference had no effect on speech onset in either group. We did not observe a change in network metrics in the control group due to the emotional challenge. It has been proposed that elderly individuals tend to neglect negative information in favor of positive information in cognitive processing. This is known as the positivity effect (Carstensen & Mikels, 2005). We argue that stable network metrics after a negative emotional challenge might provide a network explanation for this positivity effect. This also fits with a study by Eldesouky and English (2018), who report improvements of emotional regulation over the lifespan. The patient network was not as stable after an emotional challenge. We observed increases in mean nodal degree and mean nodal strength, both measures of network integration. Global efficiency, another integration measure, was slightly increased but did not pass the Bonferroni-corrected significance threshold. The patient network was better at efficiently distributing information throughout the network in an emotional situation than in a neutral one. Nonetheless, all metrics were still much lower than in the control group.

While we did not observe changes in network metrics within the control group, there was a distinct change in network communication patterns in the control network, as in the disgusted condition, the network lost all connector hubs. This indicates decreased intermodular communication, leading to more modularized processing. It has been shown that affective processing can lead to increased network segregation compared to neutral processing (Zhang, Li, & Pan, 2015). It could be the case that when faced with a potentially dangerous stimulus (a disgusted face), communication between modules is neglected to facilitate fast intramodular processing. While we observed distinct changes in network communication patterns due to emotion processing in the control group, the patient network did not change in this regard. As it did not exhibit any connector hubs in the neutral condition, we were not able to find a reduction in connector hubs as seen in controls. Therefore, it seems, emotional priming could not further modularize processing. This could explain PD-related behavioral differences in emotional priming reported previously. For instance, Castner et al. (2007) found that while healthy controls exhibited a delayed reaction to negative primes, this was not the case in unmedicated PD patients. If affective priming does not alter information processing in PD patients like it does in healthy controls, as seen in our analysis, one would not expect to find comparable behavioral changes.

Although we observed the loss of all connector hubs after an emotional challenge in the control group, network pillars remained mostly stable. Only one region (right Broadman area 3b) lost its pillar status, while two more regions became network pillars (right cerebellar lobule VI and right lingual gyrus). It seems that regions which act as key communicative players in the network are robust against emotional distraction. This is in line with our behavioral results, which showed no differences in speech parameters across emotional conditions. Our supplementary generalized linear model (GLM) analysis (see Supporting Information) also found no condition differences. Taken together, this indicates that even though emotional distraction influences how the brain processes information on a network level, these changes can be compensated and are not reflected in behavioral outcome. In the patient network, most pillars, too, remained stable after emotional distraction. However, four regions lost pillar status, and three became pillars. Those seven regions were all areas that did not reach pillar status in the control network and their change in pillar status in the patient network could therefore be attributed to its more random network organization.

Our study illustrates SPN alterations in PD. However, there are limitations to this study that require caution when interpreting its results. First, while our sample size is similar to that of previous studies (Fuertinger et al., 2015; Meunier, Achard, Morcom, & Bullmore, 2009; Simonyan & Fuertinger, 2015; Tessitore et al., 2012), it might be too small to reliably reflect population effects. Second, the sample size is not balanced between the patient and control groups, which may lead to a higher signal-to-noise ratio in the control data. However, the impact of the signal-to-noise ratio on the final results is likely limited by the employed percolation thresholding approach, which excluded spurious network connections by computing each group’s network separately and retaining the maximum information concerning community detection, even in the presence of noise and intersubject variability (Bordier et al., 2017). Third, the sex distribution differs between groups, with the patient group including more males, while the control group included more females. Future studies should examine larger and better sex-balanced patient/control groups. In spite of these three concerns, our results are in line with the results of resting-state studies in PD (Göttlich et al., 2013; Kim et al., 2017; Olde Dubbelink et al., 2014; Skidmore et al., 2011). Another limitation concerns the study paradigm. Previous research reports that PD patients have difficulties in perceiving emotional facial cues (Aiello et al., 2014; Castner et al., 2007; Iyer et al., 2019; Wagenbreth et al., 2019). This could make them less susceptible to emotional distraction, if they do not recognize the distractor. To account for this, we ran a pilot study to ensure that patients were able to recognize facial emotion expression in this paradigm. Our analysis of emotional scores also showed that patients and controls were able to discriminate neutral from disgusted faces. A further limitation refers to the complex task design combining visual and motor elements. We chose picture naming as it is a well-establish paradigm involving all levels of speech processing. However, it does not only result in speech-related activation. Furthermore, the emotional priming component made the paradigm even more complex. Yet, our results were well in line with various other studies. We therefore argue that this paradigm is well suited to provide a holistic approximation of real-life speech processing.

## CONCLUSION

Using a graph-theoretical network analysis, we determined impaired global efficiency and metrics of integration and segregation of the SPN in PD compared to healthy controls. We were able to characterize changes in the SPN in three ways. First, we showed the loss of nodes critically involved in intermodular network communication (connector hubs) with a simultaneous increase in the number of critical nodes for intramodular communication (provincial hubs) in PD. Second, we showed that in healthy older adults the key nodes of network information flow (network pillars) are mainly located in the associative cortices, probably for higher order information integration. In PD patients, these pillars are also located in the sensory and motor cortices. Third, an emotional challenge to the SPN did not change network metrics and showed only small changes in network pillar organization in healthy older persons. In contrast, in PD network metrics changed and network pillars further shifted from associative cortices to primary sensory and motor cortices, potentially as a compensatory mechanism in order to maintain information integration in a complex speech-processing task.

## ACKNOWLEDGMENTS

This experiment was realized using Cogent 2000 developed by the Cogent 2000 team at the FIL and the ICN and Cogent Graphics developed by John Romaya at the LON at the Wellcome Department of Imaging Neuroscience. The authors thank Peter Sörös for his help and valuable input concerning data analysis and interpretation.

## SUPPORTING INFORMATION

Supporting information for this article is available at https://doi.org/10.1162/netn_a_00310.

## AUTHOR CONTRIBUTIONS

Jana Schill: Conceptualization; Formal analysis; Methodology; Software; Visualization; Writing – original draft; Writing – review & editing. Kristina Simonyan: Funding acquisition; Methodology; Writing – review & editing. Simon Lang: Data curation; Writing – review & editing. Christian Mathys: Conceptualization; Writing – review & editing. Christiane Thiel: Supervision; Writing – review & editing. Karsten Witt: Conceptualization; Funding acquisition; Methodology; Project administration; Supervision; Writing – review & editing.

## FUNDING INFORMATION

Kristina Simonyan, Foundation for the National Institutes of Health (https://dx.doi.org/10.13039/100000009), Award ID: R01NS088160. Kristina Simonyan, Foundation for the National Institutes of Health (https://dx.doi.org/10.13039/100000009), Award ID: R01DC011805. Kristina Simonyan, Foundation for the National Institutes of Health (https://dx.doi.org/10.13039/100000009), Award ID: R01DC012545. Kristina Simonyan, U.S. Department of Defense (https://dx.doi.org/10.13039/100000005), Award ID: W911NF1810434. Kristina Simonyan, Amazon Web Services (https://dx.doi.org/10.13039/100008536). Kristina Simonyan, Mass General Brigham Innovation.

## TECHNICAL TERMS

Graph-theoretical network analysis: Model-free, data driven network analysis approach that describes the brain in form of a graph.
Functional connectivity: Statistical association between regions’ activation time series.
Hub analysis: Analysis of the communication patterns within the network, focusing on the most connected nodes (hubs).
Connector hub: A hub that is predominantly connected to nodes outside of its own module.
Provincial hub: A hub that is predominantly connected to nodes within its own module.
Connectivity matrices: *N* × *N* matrices containing the measure of connectivity between all pairs of the *N* regions of interest.
Normalized mutual information: Measure of information shared between two time series.
Percolation thresholding: Approach that determines the threshold at which the largest network component starts breaking apart.
