# Supplementary material for: Parkinson’s disease speech production network as determined by graph-theoretical network analysis
Source: Netw Neurosci. 2023 Jun 30;7(2):712–30. doi: 10.1162/netn_a_00310 (PMC10312286; doi:10.1162/netn_a_00310)
Supplement: Supplementary file 1 [file netn-7-2-712-s001.pdf]

Supplementary Material: Task Activation Analysis

Methods

For the general linear modelling analysis, non-brain tissue was removed from the structural images with ANTs software. The rest of the analysis was carried out in FSL (http://www.fmrib.ox.ac.uk). First, artefacts were removed from the fMRI data by running a probabilistic independent component analysis (ICA) as implemented in MELODIC (Multivariate Exploratory Linear Decomposition into Independent Components, part of FSL) Version 3.15. Then, FSL FEAT (fMRI Expert Analysis Tool) was run with the following settings: automatic brain extraction of the functional images using BET (Brain Extraction Tool), spatial smoothing with a 6mm full-width-at-half-maximum Gaussian kernel, registration to structural images and transformation into MNI standard space. For each participant, the two runs were first analyzed separately and then combined in a higher-level analysis. Then, a group level analysis was performed on the combined runs. A generalized mixed effects analysis was run using FLAME (FMRIB's Local Analysis of Mixed Effects) stage 1 and 2 with $Z>3.1$ and a corrected cluster significance threshold of $p<0.005$. Group differences were assessed by unpaired t-tests within FSL. Cluster location was determined according to the Harvard-Oxford Cortical and Subcortical Structural Atlases. For regions outside of these atlases, the MNI Structural Atlas was used.

Results

The paradigm resulted in wide-spread task-related activation in both groups (Figure S1, Table S1). There were no statistically significant activation differences between groups nor between conditions.

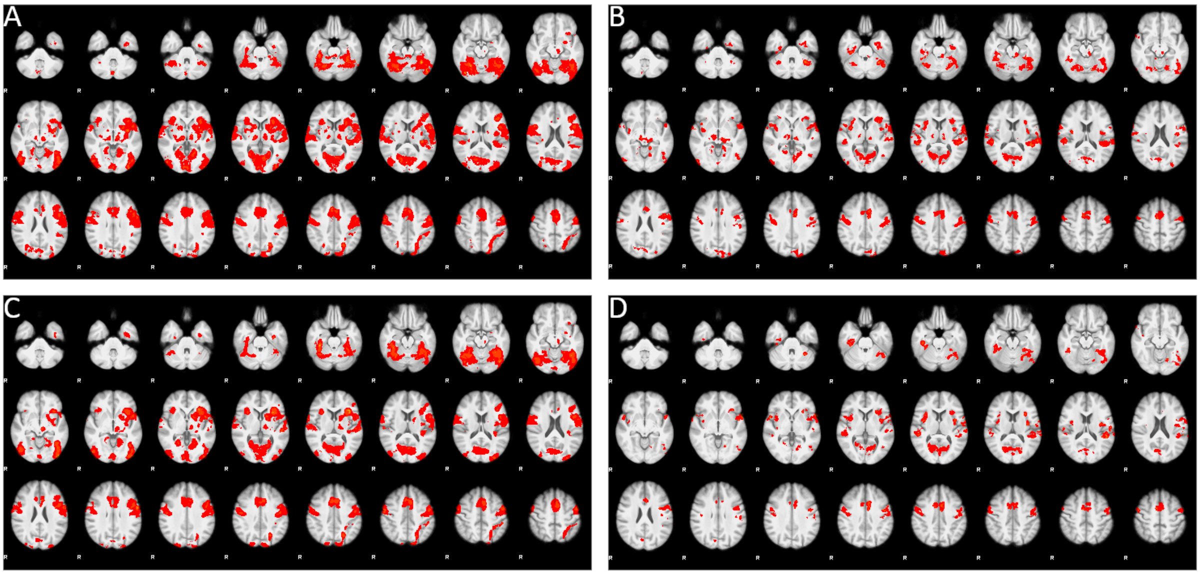

Fig. S1: Task activation maps. The paradigm resulted in wide-spread activation in both groups. A) Control group, neutral condition. There were seven clusters with maxima in the right inferior lateral occipital cortex, left inferior frontal gyrus (pars opercularis), right frontal orbital cortex, left paracingulate gyrus, right cerebellum, left and right thalamus. B) Patient group, neutral condition. There were eleven clusters with maxima in left lateral occipital cortex (inferior division), left planum temporale, left cingulate gyrus (anterior division), right inferior frontal gyrus (pars opercularis), right precentral gyrus, left brain stem, right postcentral gyrus, right cerebellum, right lateral occipital cortex (inferior division), left insular cortex and left parahippocampal gyrus (anterior division). C) Control group, disgusted condition. There were six clusters with maxima in the left temporal occipital fusiform cortex, left insular cortex, left cingulate cortex (anterior division), right precentral gyrus, right frontal orbital cortex and right cerebellum. D) Patient group, disgusted condition. There were seven clusters with maxima in the left central opercular cortex, left occipital fusiform gyrus, left cingulate gyrus (anterior division), right temporal pole, right precentral gyrus, right temporal occipital fusiform cortex and right superior temporal gyrus (posterior division).

Table S1: Brain activation.

Area of activation	x (mm)	y (mm)	z (mm)	Z value	Number of voxels
**Control Neutral**					
Right Lateral Occipital Cortex (inferior division)	52	-68	-8	9.4	15372
Left Inferior frontal Gyrus (pars opercularis)	-48	10	20	11.3	9360
Right Frontal Orbital Cortex	32	28	4	8.52	4290
Left Paracingulate Gyrus	-12	20	38	5.93	3562
Right Cerebellum	4	-78	-38	6.28	415
Left Thalamus	-10	-18	4	7.74	405
Right Thalamus	8	-18	0	6.92	345
**Control Disgusted**					
Left Temporal Occipital Fusiform Cortex	-32	-50	-18	9.63	13099
Left Insular Cortex	-30	28	2	8.56	8131
Left Cingulate Cortex (anterior division)	-2	8	30	7.46	3399
Right Precentral Gyrus	66	2	10	7.89	2523
Right Frontal Orbital Cortex	42	26	-2	6.07	384
Right Cerebellum	22	-74	-50	6.37	278
**Patient Neutral**					
Left Lateral Occipital Cortex (inferior division)	-48	-66	-12	8.23	5098
Left Planum Temporale	-62	-26	12	8.83	3173
Left Cingulate Gyrus (anterior division)	-10	8	34	8.05	2050
Right Inferior Frontal Gyrus (pars opercularis)	52	12	0	6.69	809
Right Precentral Gyrus	38	-10	38	5.97	769
Left Brain Stem	-10	-28	-8	6.18	676
Right Postcentral Gyrus	62	-12	20	6.56	592
Right Cerebellum	20	-70	-46	6.53	365
Right Lateral Occipital Cortex (inferior division)	52	-78	-4	7.06	338
Left Insular Cortex	-32	18	4	6.48	310
Left Parahippocampal Gyrus (anterior division)	-30	-4	-30	4.92	280
**Patient Disgusted**					
Left Central Opercular Cortex	-50	6	-2	7.04	2952
Left Occipital Fusiform Gyrus	-20	-66	-12	6.45	2234
Left Cingulate Gyrus (anterior division)	-4	8	42	8.07	1981
Right Temporal Pole	60	10	-2	7.11	718
Right Precentral Gyrus	54	4	42	6.33	577
Right Temporal Occipital Fusiform Cortex	46	-46	-22	6.04	499
Right Superior Temporal Gyrus (posterior division)	46	-32	2	5.79	338

Note: Coordinates are given in MNI space and refer to the voxel with the maximal Z value of a cluster.
